# The Interaction of Cellulose Thin Films With Small Organic Molecules—Comparability of Two Inherently Different Methods

**DOI:** 10.3389/fchem.2021.769022

**Published:** 2021-11-19

**Authors:** Lisa Hoffellner, Elias M. Henögl, Patrick Petschacher, Robert Schennach, Erich Leitner

**Affiliations:** ^1^ Institute of Analytical Chemistry and Food Chemistry, Graz University of Technology, Graz, Austria; ^2^ CD-Laboratory for Mass Transport Through Paper, Graz University of Technology, Graz, Austria; ^3^ Institute of Solid-State Physics, Graz University of Technology, Graz, Austria

**Keywords:** cellulose films, adsorption, desorption, solid-phase microextraction, temperature-programmed desorption

## Abstract

Paper is the material of choice for a large range of applications because it has many favorable environmental and economic characteristics. Especially in the packaging sector of dry goods and food products, paper has found unique applications. For that purpose, it has to fulfill certain requirements: Primarily it should protect the packaged goods. In order to ensure the compliance of a paper packaging, its interactions with the packaged goods should be investigated. Therefore, it is of utmost importance to understand how the paper interacts with chemicals of different nature and what factors influence these interactions—be that the nature of the paper or the characteristics of the substances. In this study, we investigated the surface interactions of cellulose thin films with n-decane and deuterated methanol using two different analytical methods: headspace solid-phase microextraction with gas chromatography and flame ionization detection (HS-SPME-GC/FID) and temperature-programmed desorption (TPD). Cellulose thin films were characterized with contact angle and FT-IR measurements and successfully applied as model systems for real paper samples. Regarding the interactions of the cellulose films with the model compounds, the two inherently different methods, HS-SPME-GC/FID and TPD, provide very comparable results. While the nonpolar n-decane was readily released from the cellulose films, the polar model compound deuterated methanol showed a strong interaction with the polar cellulose surface.

## Introduction

The need for environmentally friendly packaging materials is greater than ever before, and the demand to produce packaging solutions that fulfill both the environmental and economic aspects is still of major importance. Paper is a material that has many favorable characteristics, and it is commonly used in many different fields of applications. Also for packaging applications, it fulfills many of the desired environmental aspects. It is a sustainable, biodegradable material with excellent mechanical properties and excellent printability. Furthermore, it is economically very attractive. For the packaging of dry goods and food products, paper is already the prime choice. However, paper has to fulfill certain requirements to be used in food packaging applications. The aroma of the packaged food must be maintained inside the food, and the packaging must protect the food from the environment. As paper is an inherent porous and heterogeneous material, its use in packaging solutions can also be challenging. There should be no transfer of unwanted chemicals between the packaging and the packaged goods. Therefore, migration and permeation rates must be within legal requirements and regulations. The requirements for articles and materials intended to come into contact with food are stated in the EU Regulation 1935/2004 (Regulation (EC) 1935/2004, 2004). As there are no specific measures for paper-based food contact materials (FCMs), tests and migration limits given in the Regulation (EC) 10/2011 for plastic materials and articles that come into contact with food are often used for paper-based FCMs (Commission Regulation (Eu) No 10/2011, 2011). However, as paper is a very complex three-dimensional network with pores of different sizes (Axelsson and Svensson, 2010; Borodulina et al., 2016), it is of utmost importance to understand transfer phenomena through the paper matrix. Therefore, it is crucial to understand how the paper network interacts with different chemicals, with varying polarities and sizes. To gain a better knowledge on the interdependence of transport mechanisms and the paper matrix, the interaction of paper surface with chemical compounds should be investigated. Currently, the properties of the pore network itself and the relations between the pore network and governing transport processes are still poorly understood. Especially for paper, this knowledge can be of the highest value, as the properties of paper can be adjusted to some extent by chemical or physical treatment during the production process. For example, to increase the hydrophobicity and, in turn, the printability of a paper, it is often sized or coated with different chemical agents. Therefore, the surface properties of the paper also might change, which, in turn, might influence its interaction with various substances. With fundamental knowledge on the transport processes and how different conditions, such as the nature of the paper, can influence this transport, the production of tailor-made packaging solutions for various applications can be facilitated.

The aim of our studies was to provide analytical methods to investigate the interactions of cellulose surfaces with small molecules from the gas phase under equilibrium conditions and far from equilibrium. Therefore, we developed two inherently different methods to study the adsorption and desorption processes of selected model compounds from cellulose films. On the one hand, we used headspace solid-phase microextraction with gas chromatography and flame ionization detection (HS-SPME-GC/FID), which is a well-established and frequently used method to investigate volatile compounds in different kinds of samples but usually not used to investigate surface interactions. On the other hand, we used temperature-programmed desorption (TPD) to investigate the adsorption under nonequilibrium conditions. As with TPD, it is impossible to use paper samples; therefore, cellulose thin films were used as model systems (Henögl et al., 2019). The use of thin films enables for the first time a direct comparison of TPD and SPME-GC/FID results. In both methods, we investigated the adsorption and desorption of n-decane and deuterated methanol from the cellulose thin films. We demonstrated that both methods are complementary and the results show a very comparable adsorption/desorption behavior of the compounds on the cellulose films. This shows that the investigation of the adsorption phenomena can successfully be extended to GC-based methods, even for thin film samples.

## Materials and Methods

Both n-decane (Sigma Aldrich, purity ≥99%) and deuterated methanol (methanol-d4) (Sigma-Aldrich, purity ≥99%, 99.96 atom % D) were used without further purification.

Trimethylsilylcellulose (TMSC) (TITK, Thüringisches Institut für Textil-und Kunststoff-Forschung e.V, Germany) was dissolved in toluene. TMSC in toluene solution (10 mg/ml) was used for drop casting.

Alkenyl succinic anhydride (ASA) (Kemira, FennoSize AS 1000) was suspended in H_2_O at a ratio of 1:250,000.

Drop casting was done by dropping (40 ± 4) µl of the TMSC solution onto a polished stainless steel substrate (1 × 1 cm) and subsequent drying at atmospheric conditions for at least 2 hours. The resulting films have a convex form, ranging from 3 μm at the edges to 12 µm in the center. The areal mean value is about 5–6 μm, and the cross-sectional mean value is about 8 µm. These values were measured using profilometry.

The TMSC films were regenerated via the gas phase with 10%_vol_ concentrated HCl (37%) in H_2_O for 20 min or 60 min. Therefore, six films next to seven small drops (ca. 5 µl) of HCl were placed in a 9-cm-diameter closed Petri dish.

Sizing: Drop-casting ASA dilution was performed in two steps: addition of 160 µg ASA in 40 g of H_2_O and shaking vigorously for several minutes to form an emulsion. 100 µl of the emulsion was dropped onto the thin film and dried in laboratory air. No dispersing agents were used. Drying was performed in laboratory air under the fume hood for at least 6 h before being installed into the vacuum chamber for TPD. Final drying occurred during the pump down cycle under increasing vacuum conditions for 7 days with a final pressure of about 1 × 10^–9^ mbar. For HS-SPME-GC/FID measurements, the films were dried for 4–7 days in laboratory air under the fume hood before measurement. One should note that the amount of ASA added onto the regenerated cellulose film corresponds to approximately 1 kg ASA per ton of thin film material. This ratio is commonly used in the paper-making process.

Resulting cellulose thin film samples: TMSC, cellulose 20 min regenerated, cellulose 60 min regenerated, and cellulose 60 min regenerated + ASA.

The HS-SPME-GC/FID experiments were, as the TPD experiments, used to investigate the interactions of the cellulose films with n-decane and methanol-d4. Therefore, the same samples as those for TPD measurements (Table 1) were prepared and analyzed.

**TABLE 1:** Contact angles (CA) and number of measurements (N) (Henögl, 2021).

Sample	CA [°]	N
Stainless steel	84 ± 2	8
TMSC	100 ± 1	8
Cellulose 20-min regeneration	54 ± 3	6
Cellulose 60-min regeneration	49 ± 8	5
Cellulose 60-min regeneration + ASA	106 ± 1	8

Unlike in the TPD studies, which were carried out under nonequilibrium conditions, the HS-SPME-GC/FID measurements were performed under equilibrium conditions. In the experimental setup, the thin film samples were placed in 20 ml headspace crimp vials with the thin film side looking up. 20 nl (14.6 µg) of n-decane or 20 nl (17.8 µg) of methanol-d4 was applied by touching the tip of the syringe needle on the glass wall in the upper region of the vial to prevent direct contact to the thin film sample. To avoid losses by evaporation, the analytes were added directly into the closed vial using a 1-µl syringe (Hamilton 7001KH, United States). To avoid direct interaction of the analytes with the sample, the compounds were left to be adsorbed via the gas phase only. The closed vials were equilibrated for 24 h at room temperature before measurement. Determinations were carried out in triplicate.

The headspace above the sample was extracted using HS-SPME with a 2-cm stable flex 50/30 µm DVB/Carboxen/PDMS fiber (Supelco Inc., United States). The conditions were as follows: 70°C incubation temperature, 15 min extraction time, and 6 min desorption time. An Agilent 6890 series gas chromatograph equipped with an autosampler with an SPME option (CTC Analytics, Switzerland) and a flame ionization detector was used (Agilent, United States). The analytes were separated on a Zebron ZB-5 MS Plus column (30 × 0.25 mm; 0.25 µm) from Phenomenex (United States). The following GC parameters were applied: detector temperature 320°C, injector temperature 270°C, and injection mode splitless. The column temperature program was as follows: 50°C (1 min), from 50°C at a rate of 8°C/min to 270°C (1 min). Helium was used as the carrier gas with a constant flow rate of 1.6 ml/min.

The amount of adsorbed or desorbed compounds was calculated using the following approach: As the reference point, an identical experimental procedure with a vial containing only the uncoated stainless steel substrate and the same amount of analyte was used. The chromatographic peak area of the compound of interest as obtained after the HS-SPME-GC-FID analysis of the headspace exposed to the uncoated substrate was measured. This peak area refers to 0% adsorption and is used to calculate the desorbed amount from the thin film samples.

Atomic force microscopy (AFM) measurements were performed using a Nanosurf Easyscan 2 (Switzerland) using the same kind of samples. The data were acquired using Easyscan 2 control software from Nanosurf. Data analysis and visualization were performed using the free software Gwyddion (gwyddion.net). For the samples presented here, an AFM head with a maximum field of view of 70 × 70 µm and a maximum height change of 14 µm was used. As AFM tips, PPP-CONTR cantilevers from Nanosensors (Switzerland) were used. These cantilevers have a resonance frequency of 13 kHz, a force constant of 0.2 N/m, a length of 450 μm, a mean width of 50 µm, and a thickness of 2 µm. For comparisons between the different films, all AFM images are provided with a 50 × 50-µm resolution.

Profilometry measurements were carried out using an Alpha-Step D-500 profilometer from KLA Tencor. The films were measured using a step-up and step-down procedure with a stylus speed of 0.1 mm/s.

Contact angle measurements were carried out using a CAM200 optical contact angle meter (KSV). A test drop of 5 +− 1 µl of demineralized water was used. The dropping method was drop forming, approaching, lay down, needle retraction, and recording. The contact angle was calculated using a Young–Laplace fit.

FT-IR measurements were performed using a Bruker IF66v/s (Bruker, Germany) spectrometer with specular reflectance at an angle of 78° under rough vacuum. The resolution was 4 cm^−1^. A clean stainless steel sample was used as reference.

Temperature-programmed desorption (TPD) was carried out in a home-built high-vacuum system described in detail earlier (Henögl et al., 2019). For the TPD experiments, a stainless steel substrate coated with a cellulose thin film was mounted onto the vacuum chamber. For all experiments, we pumped the system down to a background pressure of about 1 × 10^–9^ mbar. To bring the methanol-d4 or the n-decane into the gas phase, we used their liquid vapor pressure at room temperature. The used liquid (either n-decane or methanol-d4) was stored in a glass reservoir connected to the vacuum chamber via a needle valve. After the liquids were initially put in the reservoir, air was pumped out of the gas dosing system by several freeze, pump, and thaw cycles. To avoid cross contamination and false-positive results, per deuterated methanol was used due to the differences in the fragmentation pattern in the mass spectra. For the TPD experiments, first, the sample was cooled down to –80°C. Then, n-decane or methanol-d4 was brought into the gas phase by opening the needle valve and raising the pressure to about 5 × 10^–6^ mbar for 6 min. After the exposure, we waited until the background pressure was reached again. Then, the sample was resistively heated at a linear heating rate of 1 °C/s until a maximum temperature of 600°C was reached. For each experiment, a new sample was used.

## Results and Discussions

In this section, first the properties of the samples are discussed. Then, the TPD results and the HS-SPME-GC/FID results are presented and discussed.

### Atomic Force Microscopy

**FIGURE 1:**
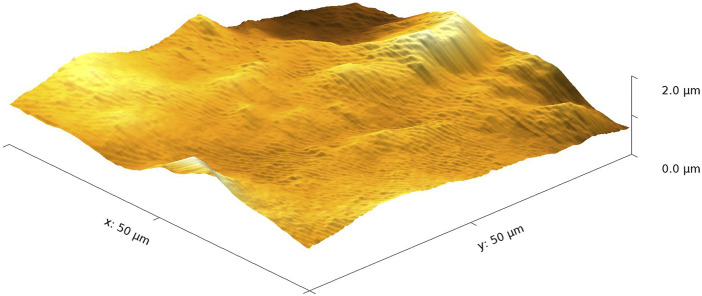
AFM measurement of a drop-casted TMSC film.

While spin-coated TMSC films have been widely investigated in the literature, (e.g., Kontturi and Spirk, 2019 and references therein), drop-casted films have received much less attention. In Figure 1, an AFM image of a drop-casted TMSC film is shown. The thickness of the films was measured using profilometry, and this led to a cross-sectional mean value of about 8 µm (areal mean of about 5–6 µm). The root mean square (RMS) roughness is 236 nm. The large height differences (the maximum height is 1.7 µm) are due to the drop-casting, as evaporation of the solvent using this technique is much slower than that using the spin-coating technique. But in the nanometer scale, the roughness is small, as is known from spin-coated TMSC films.

**FIGURE 2:**
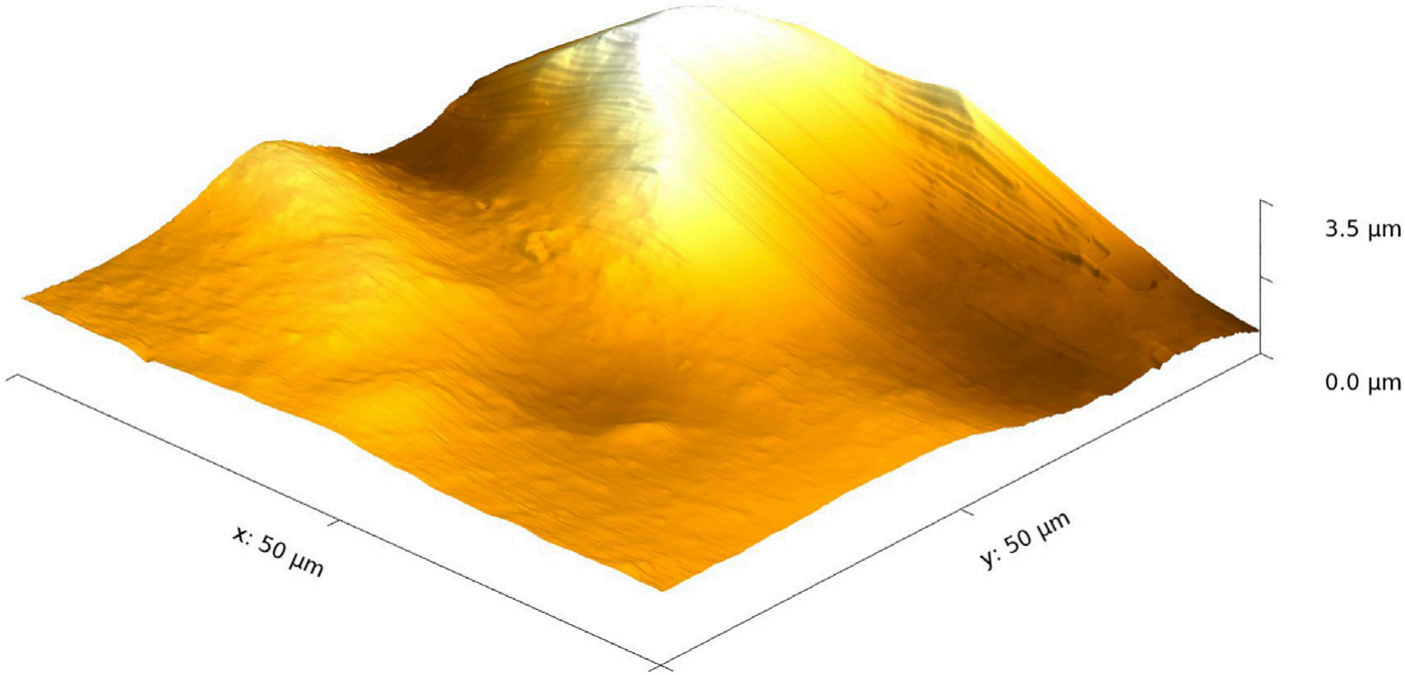
AFM image of an ASA-modified TMSC film.

The TMSC film modified with ASA looks similar to the pure TMSC film (see Figure 2). At a closer look, one notices a much larger feature with a maximum height of about 3.54 µm. Such large islands were only found on ASA-modified TMSC films, indicating that ASA does not spread evenly on the hydrophobic TMSC surface. Maybe ASA formed micelles in the suspension in water, and this might influence the ASA–surface interaction. The measured surface roughness increased to 572 nm on the ASA-modified film.

**FIGURE 3:**
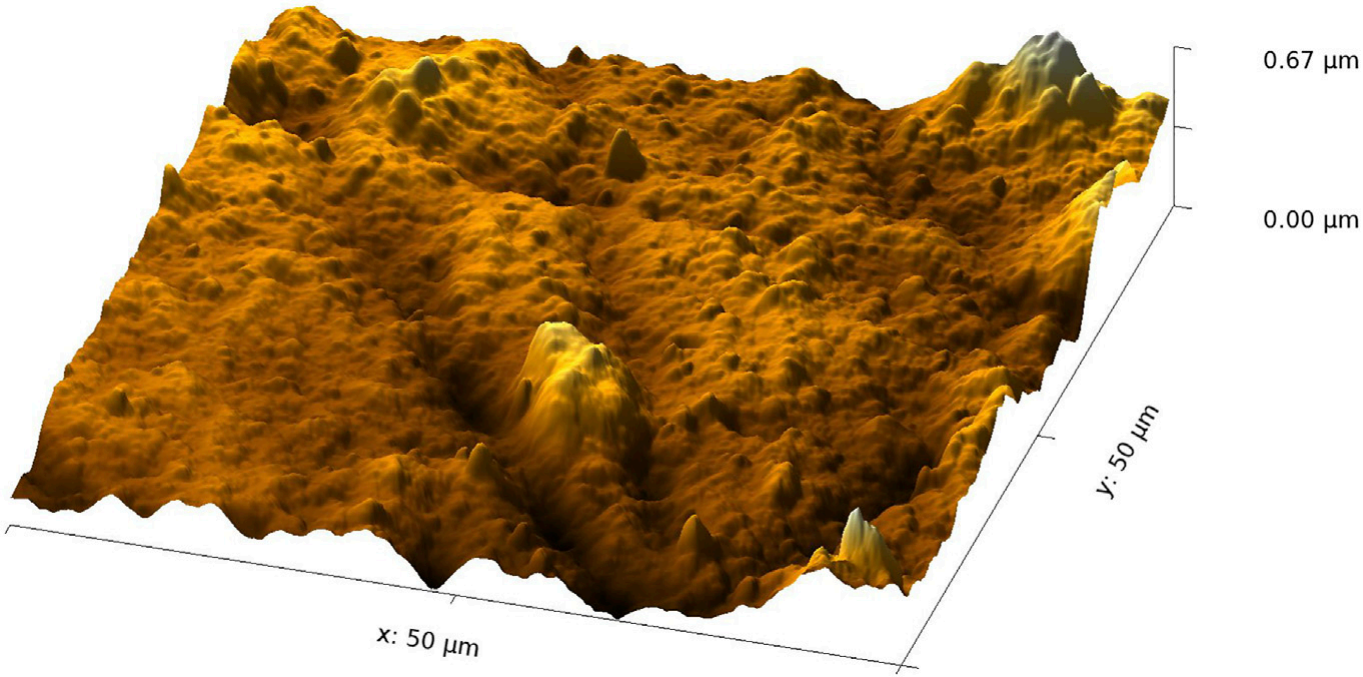
AFM image of a 20-min regenerated film.

An AFM image of the 20-min regenerated film is shown in Figure 3. The changes in the modified film compared to the TMSC film have already been reported in the literature (Kontturi et al., 2006). The film thickness decreases to a cross-sectional mean value of about 7 µm (the areal mean value is about 4–5 µm). Also, the RMS roughness decreases to about 75 nm.

**FIGURE 4:**
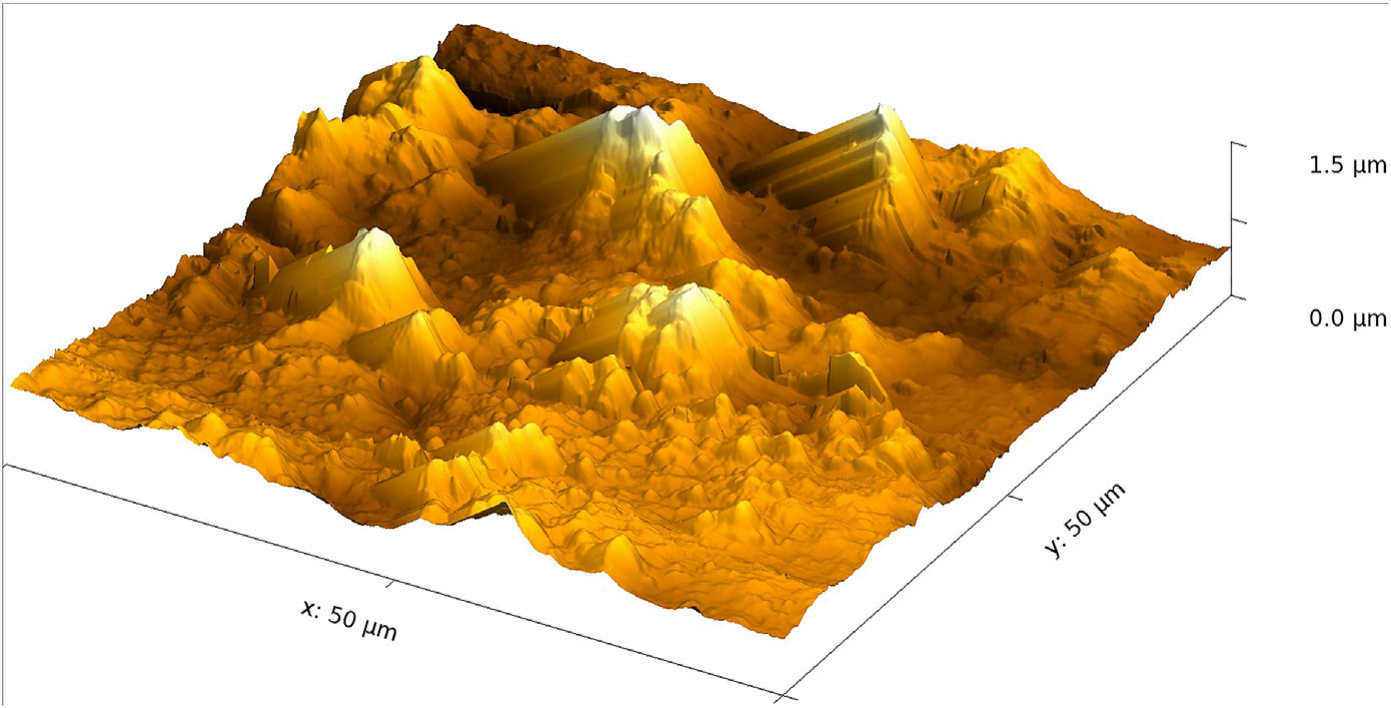
AFM image of an ASA-modified 20-min regenerated film.

When ASA is drop-casted onto the 20-min regenerated film, one notices a strongly increased surface roughness (RMS is 184 nm) and again larger islands (see Figure 4). This shows quite clearly that the drop-casted ASA has a strong tendency to form islands on the cellulosic surfaces studied here. We found very similar results for the 60-min regenerated film. The corresponding AFM images are shown in the supporting information in Supplementary Figure S1 and Supplementary Figure S2.

### Contact Angle and Polarity

**FIGURE 5:**
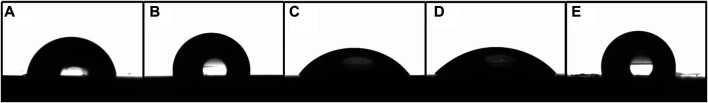
Contact angle of stainless steel substrate **(A)**, TMSC **(B)**, cellulose 20-min regeneration **(C)**, cellulose 60-min regeneration **(D)**, cellulose 60-min regeneration + ASA **(E)** (Henögl, 2021).

To obtain information about the hydrophilicity of the films, contact angle measurements were performed. As can be seen in Figure 5, there are clear differences between the different films.

The contact angle measurements are tabulated in Table1. The number of measurements is given in the right column, and the results are presented as mean values and standard deviation. One can clearly see that the drop-casted TMSC film shows a hydrophobic surface behavior with a static water contact angle of about 100°. As one would expect, this is changed to a much more hydrophilic surface, with a contact angle of around 50°, when surface regeneration is performed. Both contact angles are well in line with values reported for TMSC and regenerated cellulose in the literature (Schlemmer et al., 2018). The 60-min regenerated cellulose film sized with ASA features again a very high contact angle of 106°. This shows that even this small amount of ASA has a very significant impact on the contact angle of the thin film, comparable to the impact of a sizing on an unbleached paper. In addition, one has to note that the static contact angle with ASA is high, even though ASA forms individual islands on the surface according to the AFM results.

### FT-IR Measurements

**FIGURE 6:**
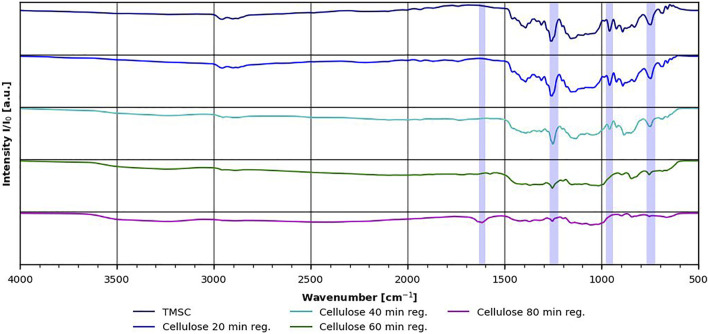
FT-IR of thin films with progressing regeneration time. The shaded areas refer to TMSC vibration, and the one around 1,620 cm^−1^ corresponds to ring-opening reactions (Henögl, 2021).

FT-IR measurements were used to follow the regeneration of the TMSC films. This was also performed earlier (e.g., Schaub et al., 1993; Mohan et al., 2011). The results can be seen in Figure 6. While the peaks corresponding to the TMS group (1,250, 748 cm^−1^) are clearly visible on the TMSC film (topmost spectrum in Figure 6) and on the 20-min regenerated film, on the 40-min regenerated film, the peaks are smaller. However, even after 60 min of regeneration, small remnants of these peaks can still be seen. This might be due to the overall film thickness, which might lead to the entrapment of the TMS group in the film even though the cellulose has been completely regenerated. This assumption is supported by the fact that the SiOC stretching band between 990 cm^−1^ and 945 cm^−1^ is not visible in the 60-min regenerated sample. After 80 min of regeneration (lowest spectrum in Figure 6), a new peak around 1,620 cm^−1^ can be seen, which is the product of cellulose ring-opening reactions. When the hydrolysis of cellulose sets in, one can safely assume that TMS hydrolysis is finished. Nevertheless, one can still see the small remnants of the TMS peaks in this spectrum.

The samples with ASA were also measured; however, no further changes could be observed. The amount of ASA on the surface was too small to be detected in the IR spectra. Therefore, we cannot say from the FT-IR results alone if ASA was covalently bound to the surface or just adsorbed.

### Temperature-Programmed Desorption Experiments

**FIGURE 7:**
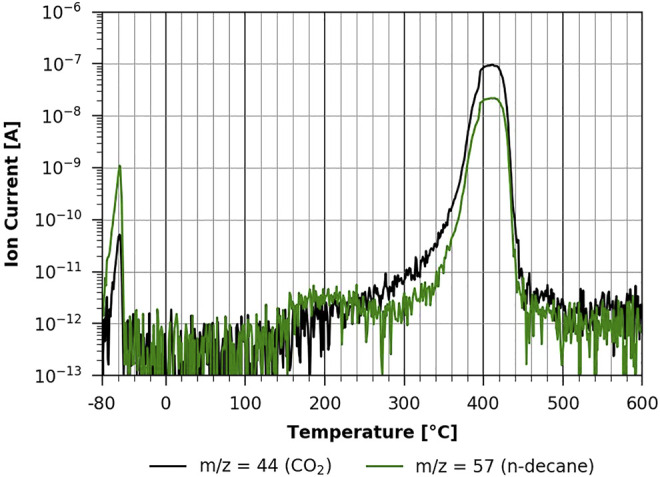
TPD of n-decane from pure TMSC (Henögl, 2021).

In this section, the TPD results are shown, first for n-decane and then for methanol-d4 desorption from different cellulosic surfaces. In Figure 7, one can see the TPD spectrum of a pure TMSC film following mass 57 as the largest fragment of n-decane and mass 44 as CO_2_, which is also a product of cellulose pyrolysis. One can clearly see the physisorbed n-decane multilayer desorption at low temperatures with a peak maximum at about −60°C. The peak and its desorption energy are well comparable with the heat of evaporation found in the literature (Majer, Svoboda and Kehiaian, 1985; Henögl et al., 2019). Since it is a multilayer desorption that is less dependent on the underlying surface, it is also quite comparable to the n-decane desorption from the stainless steel substrate, shown in the supplementary information (Supplementary Figure S3)

At a temperature of about 150°C, one can see the onset of the first phase of the TMSC pyrolysis with a broad peak centered around 200°C, followed by a large signal at around 420°C. This main desorption/pyrolysis reaction with strong CO_2_ emission can be approximated using the Redhead formula (Redhead, 1962) to an activation energy of about 190 kJ/mol and has been previously shown to stem from cellulose pyrolysis (Henögl et al., 2019). Cellulose pyrolysis was found to take place in multiple stages by Yeo et al., who also stated a first-order reaction with a comparable activation energy (Yeo et al., 2019).

**FIGURE 8:**
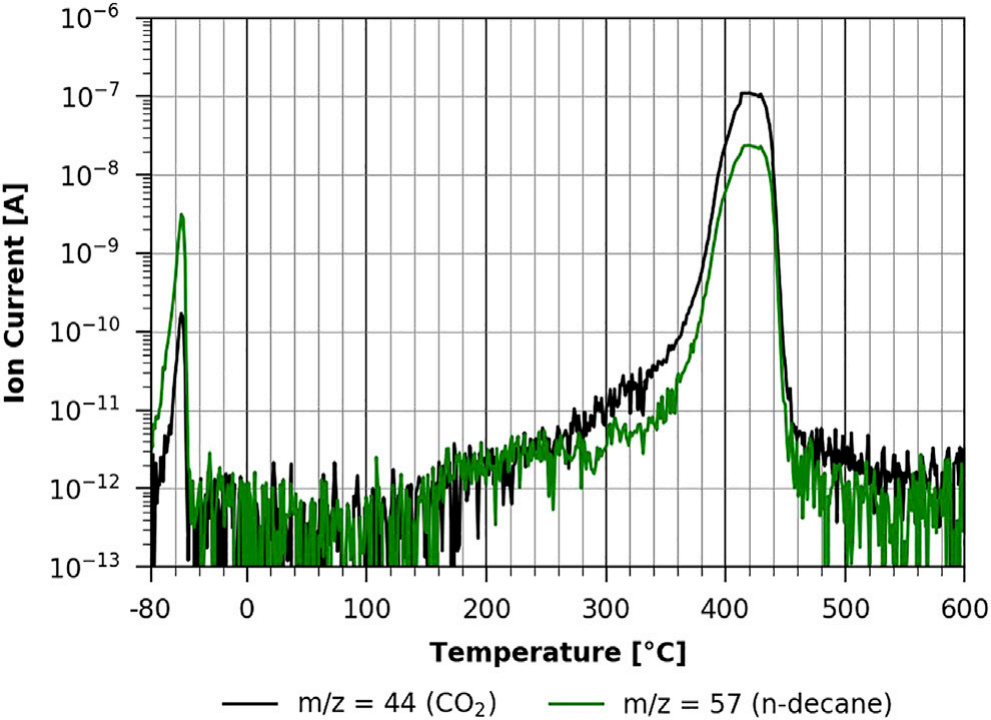
TPD of n-decane from cellulose 20-min regeneration (Henögl, 2021).

**FIGURE 9:**
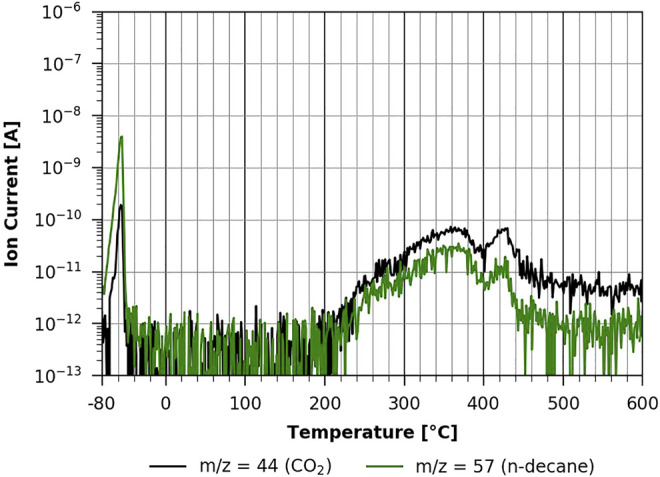
TPD of n-decane from cellulose 60-min regeneration (Henögl, 2021).

In Figure 8, one can see the same experiment conducted with the 20-min regenerated cellulose film. It is clear that both the thermal desorption of n-decane and the pyrolysis of the film are very similar to the non-regenerated case shown in Figure 7. In both cases, n-decane desorption is in the same temperature range, strongly suggesting that it is a physisorbed multilayer desorption process, as the interaction with the two different substrates is apparently the same. This is also the case with the fully regenerated cellulose film (shown in Figure 9). However, as can be seen in Figure 9, the pyrolysis peaks show a very different behavior. Volatilization due to pyrolysis only starts at around 220°C, and one can clearly differentiate two main peaks, one with a maximum at about 360°C and the other one with a maximum at about 430°C. This shows that a TMSC film and a pure cellulose film show a very different behavior during pyrolysis. The pure cellulose film coated with ASA (see Supplementary Figure S7 in the Supplementary Material) has basically the same characteristic as the one without ASA.

In all measurements with n-decane, we found that desorption occurs in the temperature range between about −70°C and −45 °C. This corresponds to an adsorption energy of about 60 kJ/mol according to the Redhead formula (Redhead, 1962). This compares well with values of the physisorption energy of n-decane in the literature of ΔH_vap_ = 58 kJ/mol interpolated to −54°C (Majer, Svoboda and Kehiaian, 1985) and $\Delta {Ε}_{des}^{mult}$ = 52.4 kJ/mol at −80°C on graphite (Paserba and Gellman, 2001).

**FIGURE 10:**
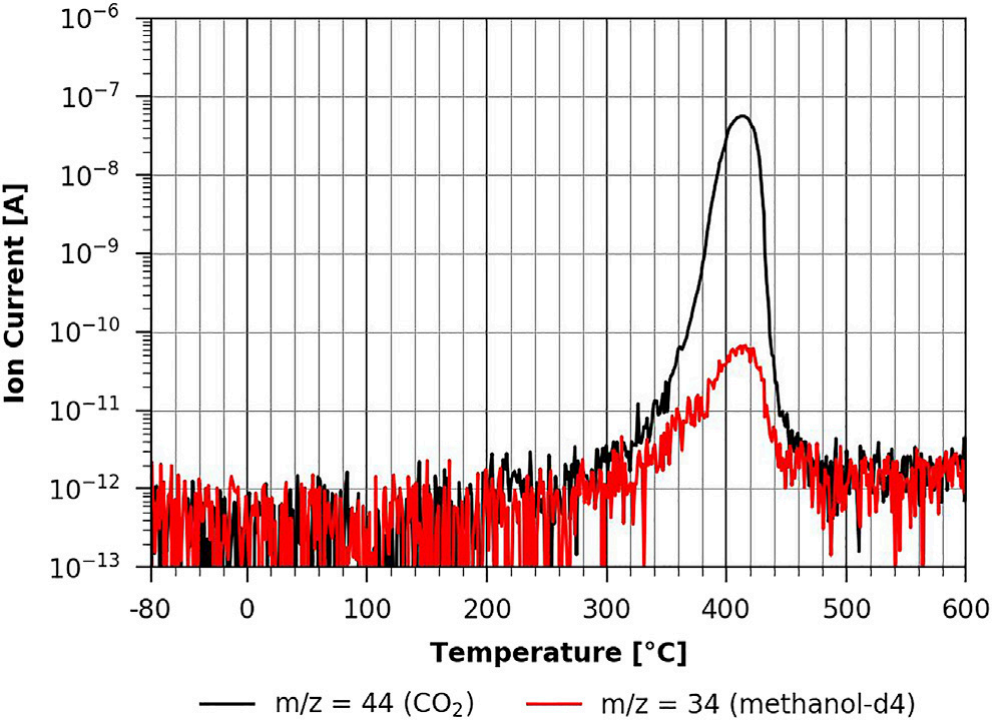
TPD of methanol-d4 from TMSC (Henögl, 2021).

In Figure 10, one can see the methanol-d4 desorption from a pure TMSC film. The TPD spectra look very similar to the n-decane case with the exception that no low-temperature desorption features can be seen. The fact that one cannot observe a multilayer desorption of methanol-d4 can be explained by its low heat of evaporation. Such a multilayer desorption has been found from graphene at −130°C by Smith, Matthiesen and Kay (2014) and is thus most likely below the here investigated temperature range.

**FIGURE 11:**
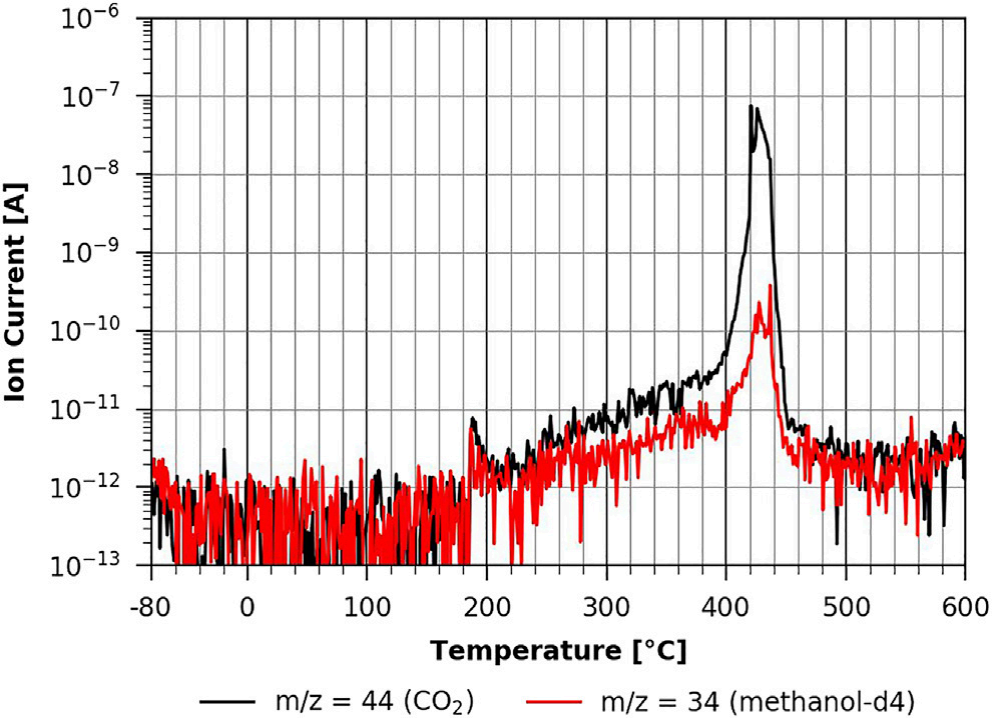
TPD of methanol-d4 from cellulose 20-min regeneration (Henögl, 2021).

**FIGURE 12:**
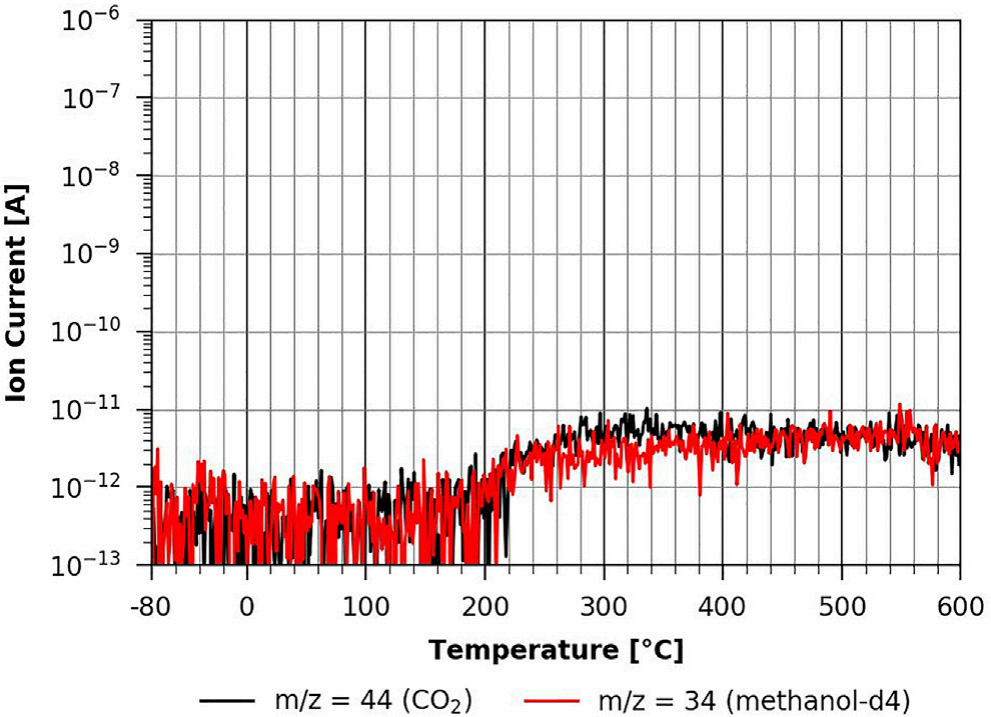
TPD of methanol-d4 from cellulose 60-min regeneration (Henögl, 2021).

However, when one studies the methanol-d4 desorption from the partially regenerated cellulose film, one can see a clear impact of methanol-d4 on pyrolysis as the corresponding peaks show a very different shape (see Figure 11). This effect gets even stronger on the fully regenerated cellulose film shown in Figure 12. Here the pyrolysis seems to be much slower and still occurring at the maximum desorption temperature of 600°C that could be reached with the current experimental setup. This suggests that we do not see a methanol-d4 monolayer desorption, since methanol-d4 is strongly bound to cellulose and apparently even takes part in cellulose pyrolysis. This would explain the significant change in the pyrolysis with methanol-d4 as compared to n-decane adsorption. This shows that the polar cellulose film strongly interacts with the polar adsorbent. Surprisingly, methanol-d4 desorption from an ASA-modified cellulose film is identical to the pure cellulose case (see Supplementary Figure S6 in the supplementary information). Apparently, there is a big difference between a TMSC film which is hydrophobic and a hydrophobic coating (such as ASA). The ASA forms small islands on the surface (see AFM results above), leaving room in between the islands for methanol-d4 adsorption. In the case of the hydrophobic TMSC film, no measurable methanol-d4 adsorption takes place.

### Adsorption/Desorption Experiments Using Headspace Solid-Phase Microextraction With Gas Chromatography and Flame Ionization Detection

**FIGURE 13:**
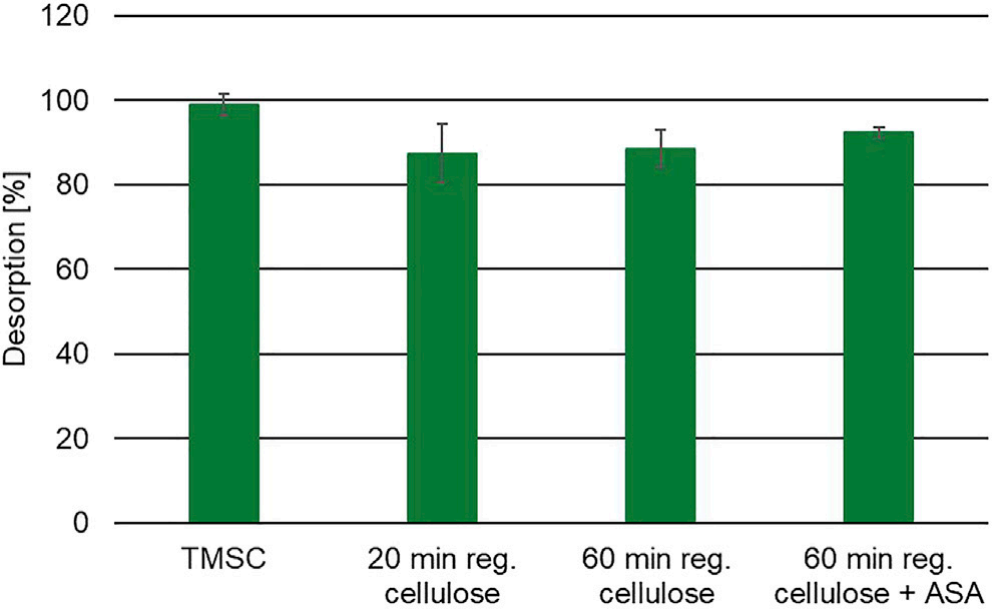
Desorption of n-decane from cellulose thin films (Hoffellner, 2021).

Figure 13 shows the desorption in percentage of n-decane from the samples investigated in our studies. As can be seen from the figure, n-decane is released from all samples. Although the desorption trend of n-decane from all samples is similar, a *t*-test analysis showed a statistically significant difference between the TMSC and the 60-min regenerated cellulose film at a confidence level of 95%. As cellulose is a very polar material, there might be no or only weak interactions with the nonpolar compound n-decane and the cellulose surface. These results are in very good agreement with those obtained during the TPD measurements. Even when the surface of the cellulose film is modified by applying the paper-sizing agent ASA, n-decane does not bind to this more hydrophobic surface. The same effect was observed in one of our previous studies. The small, nonpolar compounds were released from unsized and sized paper samples (Hoffellner and Leitner, 2020).

**FIGURE 14:**
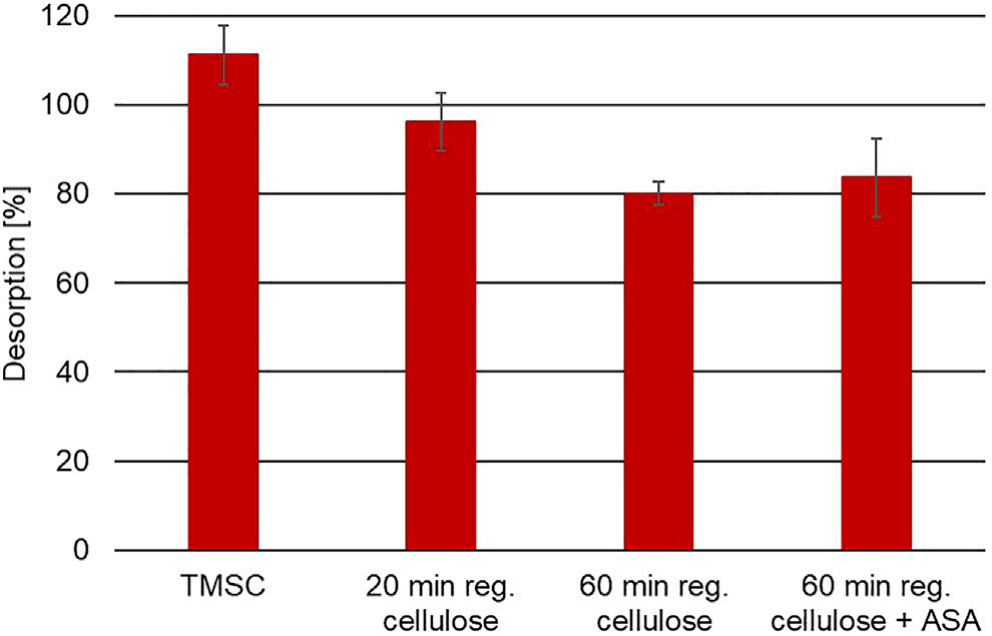
Desorption of methanol-d4 from cellulose thin films (Hoffellner, 2021).

Figure 14 shows the desorption of methanol-d4 from the cellulose thin films. It is obvious from the figure that methanol-d4 only sorbs on the more polar samples, for example, the 60-min regenerated cellulose films. According to the *t*-test analysis, there is a statistically significant difference between the TMSC film and the 60-min regenerated cellulose film at a confidence level of 95%. Although desorption from the cellulose film treated with ASA is slightly higher than desorption from the pure cellulose film, there is no significant difference in desorption between these two samples. In the TPD measurements, no monolayer desorption peak of methanol-d4 was found; in the HS-SPME-GC/FID measurements, around 20% methanol-d4 sorbed on the 60-min regenerated cellulose samples. In turn, on the polar 60-min regenerated sample, around 3.5 µg of methanol-d4 sorbed. This fact also supports the assumption of the formation of a strongly bound monolayer of methanol-d4 molecules on the cellulose film under vacuum conditions. As in TPD, only a monolayer might adsorb, especially in the investigated temperature range, and no desorption peak could be found.

Since 3.5 µg has to be considered much more than a monolayer, additional absorption and adsorption due to a higher mean population under atmospheric conditions have to be considered.

These findings are also in a good agreement with other studies in which paper samples were spiked with polar model compounds. It was shown that polar compounds strongly interact with polar paper samples (Walzl et al., 2019; Hoffellner and Leitner, 2020).

## Conclusion

In this study, the surface interactions of cellulose thin films with nonpolar and polar molecules were investigated using two different analytical techniques, TPD and HS-SPME-GC/FID. Although TPD works under nonequilibrium conditions and HS-SPME-GC/FID works under equilibrium conditions, both methods were successfully applied and provided highly comparable results. As n-decane is a nonpolar molecule, it does not interact strongly with the polar cellulose film and is readily released. The polar molecule methanol-d4 is more strongly adsorbed, a fact that can be explained by its higher affinity to the polar cellulose surface. Furthermore, it was shown that with HS-SPME-GC/FID, surface interactions of complex samples can be studied. The sizing of the regenerated thin films was sufficient to change the macroscopic contact angle of a liquid drop placed on the sample. But it seems clear that the methanol-d4 adsorption is hardly hindered by this ASA treatment, confirming the AFM results that ASA does not form a continuous film on the surface under these conditions. Finally, a strong influence of the adsorbed methanol-d4 on cellulose pyrolysis has been found.

## Data Availability

The original contributions presented in the study are included in the article/Supplementary Material; further inquiries can be directed to the corresponding authors.

## References

[B1] Axelsson M. Svensson S. (2010). 3D Pore Structure Characterisation of Paper. Pattern Anal. Applic 13 (2), 159–172. 10.1007/s10044-009-0146-1

[B2] Borodulina S. Kulachenko A. Wernersson E. L. G. Hendriks C. L. L. (2016). Extracting Fiber and Network Connectivity Data Using Microtomography Images of Paper. PHYSICS Nordic Pulp Paper Res. J. 31 (3), 469–478. 10.3183/NPPRJ-2016-31-03-p469-478

[B3] Commission Regulation (Eu) No 10/2011 (2011). Commission Cegulation (EU) No 10/2011 of 14 January 2011 on Plastic Materials and Articles Intended to Come into Contact with Food. European Union: Official Journal of the European Union.

[B4] Henögl E. Haberl V. Ablasser J. Schennach R. (2019). Adsorption and Desorption of Organic Molecules from Thin Cellulose Films. Front. Mater. 6, 1–8. 10.3389/fmats.2019.00178

[B5] Henögl E. M. (2021). Adsorption and Desorption of Organic Molecules from Thin Cellulose Films Investigated by Temperature Programmed Desorption. Master Thesis. Graz: University of Technology.

[B6] Hoffellner L. Leitner E. Leitner E. (2020). Sorption Behavior of Organic Molecules on Porous Paper Material. Cellulose Chem. Technol. 54 (5–6), 515–522. 10.35812/CelluloseChemTechnol.2020.54.52

[B7] Hoffellner L. (2021). The Interaction of the Paper Matrix with Volatile Organic Compounds. Dissertation. Graz: University of Technology.

[B8] Kontturi E. Spirk S. (2019). Ultrathin Films of Cellulose: A Materials Perspective. Front. Chem. 7, 488. 10.3389/fchem.2019.00488 31380342 PMC6652239

[B9] Kontturi E. Tammelin T. Österberg M. (2006). Cellulose-model Films and the Fundamental Approach. Chem. Soc. Rev. 35, 1287–1304. 10.1039/b601872f 17225889

[B10] Majer V. Svoboda V. Kehiaian H. V. (1985). Enthalpies of Vaporization of Organic Compounds: A Critical Review and Data Compilation. Oxford: Blackwell Scientific.

[B11] Mohan T. Kargl R. Doliška A. Vesel A. Köstler S. Ribitsch V. (2011). Wettability and Surface Composition of Partly and Fully Regenerated Cellulose Thin Films from Trimethylsilyl Cellulose. J. Colloid Interf. Sci. 358 (2), 604–610. 10.1016/j.jcis.2011.03.022 21458821

[B12] Paserba K. R. Gellman A. J. (2001). Effects of Conformational Isomerism on the Desorption Kinetics of N-Alkanes from Graphite. J. Chem. Phys. 115 (14), 6737–6751. 10.1063/1.1398574

[B13] Redhead P. A. (1962). Thermal Desorption of Gases. Vacuum 12 (4), 203–211. 10.1016/0042-207X(62)90978-8

[B14] Regulation (EC) 1935/2004 (2004). Regulation (EC) No 1935/2004 of The European Parliament and of The Council of 27 October 2004 on Materials and Articles Intended to Come into Contact With Food and Repealing Directives 80/590/EEC and 89/109/EEC.

[B15] Schaub M. Wenz G. Wegner G. Stein A. Klemm D. (1993). Ultrathin Films of Cellulose on Silicon Wafers. Adv. Mater. 5 (12), 919–922. 10.1002/adma.19930051209

[B16] Schlemmer W. Zankel A. Niegelhell K. Hobisch M. Süssenbacher M. Zajki-Zechmeister K. (2018). Deposition of Cellulose-Based Thin Films on Flexible Substrates. Materials 11 (12), 2433. 10.3390/ma11122433 PMC6316936 30513642

[B17] Smith R. S. Matthiesen J. Kay B. D. (2014). Desorption Kinetics of Methanol, Ethanol, and Water from Graphene. J. Phys. Chem. A. 118 (37), 8242–8250. 10.1021/jp501038z 24654652

[B18] Walzl A. Kopacic S. Bauer W. Leitner E. (2019). Characterization of Natural Polymers as Functional Barriers for Cellulose-Based Packaging Materials. Food Additives & Contaminants: A 36, 976–988. 10.1080/19440049.2019.1600747 30994406

[B19] Yeo J. Y. Chin B. L. F. Tan J. K. Loh Y. S. (2019). Comparative Studies on the Pyrolysis of Cellulose, Hemicellulose, and Lignin Based on Combined Kinetics. J. Energ. Inst. 92 (1), 27–37. 10.1016/j.joei.2017.12.003

