# Supplementary material for: The Interaction of Cellulose Thin Films With Small Organic Molecules—Comparability of Two Inherently Different Methods
Source: Front Chem. 2021 Nov 19;9:769022. doi: 10.3389/fchem.2021.769022 (PMC8639685; doi:10.3389/fchem.2021.769022)
Supplement: Supplementary file 1 [file DataSheet1.pdf]

Supplementary Information prepared for the following manuscript for “Cellulose”:

The interaction of cellulose thin films with small organic molecules – Comparability of two inherently different methods

Lisa Hoffellner*[1, 3], Elias M. Henögl[2,3], Patrick Petschacher[2] Robert Schennach* [2, 3], Erich Leitner[1, 3]

[1]Institute of Analytical Chemistry and Food Chemistry, Graz University of Technology, Austria; [2]Institute of Solid-State Physics, Graz University of Technology, Austria; [3]CD-Laboratory for Mass Transport through Paper, Graz University of Technology, Austria

*Corresponding authors: Lisa Hoffellner lisa.hoffellner@tugraz.at; Robert Schennach robert.schennach@tugraz.at

The AFM pictures of 60 minutes regenerated films and the 60 minutes regenerated film with ASA is shown in figures S1 and S2, respectively.

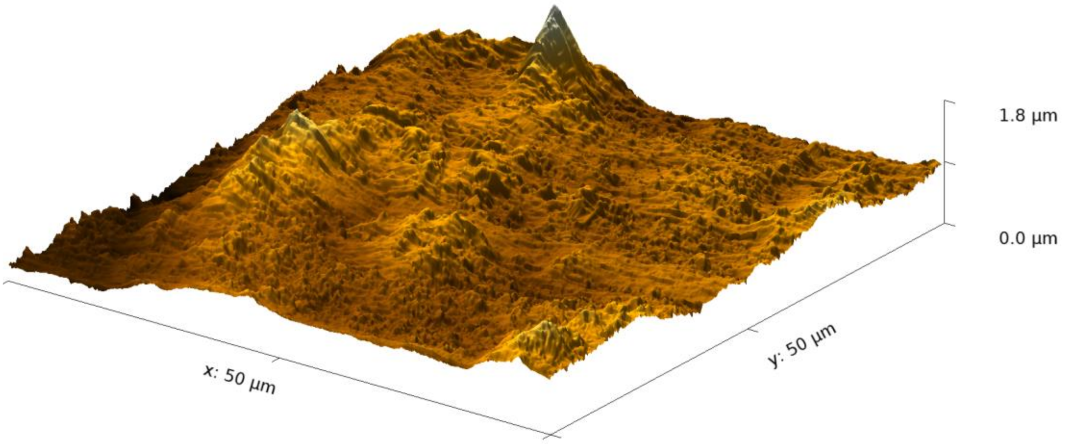

Fig S1 AFM image of a 60 minutes regenerated film.

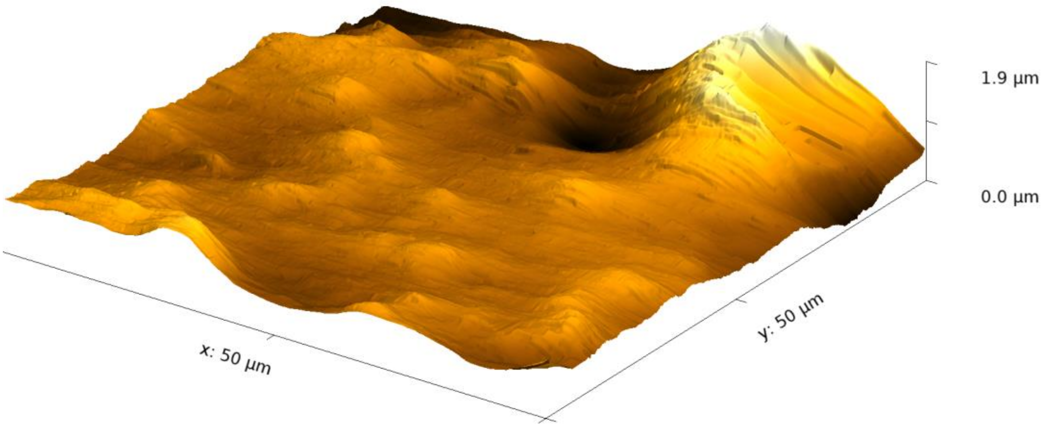

Fig. S2 AFM image of a 60 minutes regenerated film with ASA.

As blind experiments we first made a desorption experiment from a clean stainless steel substrate without additional adsorption. As can be seen in Fig. S3 there is no desorption detectable from the surface. We followed mass 44 for CO_2 desorption (product of cellulose pyrolysis) and mass 57 (largest intensity fragment of n-decane), mass 34 (fragment of methanol-d4) and mass 18 for water.

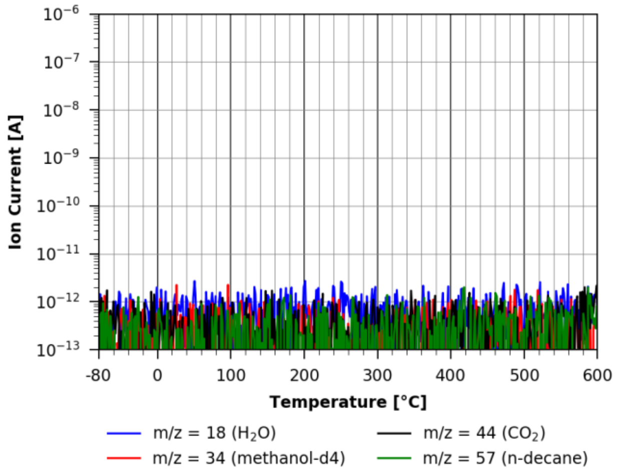

Fig. S3. Empty experiment with stainless steel substrate without adsorption (Henögl, 2020)

Second, we made a TPD experiment with n-decane on the clean stainless steel substrate. The result can be seen in Fig. S4. From Fig. S4 it is clear that n-decane adsorbs on the stainless steel substrate at about -80°C. Desorption starts immediately with the start of the temperature ramp. The peak maximum is at around -50°C. The asymmetric peak shape with the steep drop at the higher temperature side strongly suggest a 0-order desorption, implying that the n-decane was physisorbed in a multilayer on the stainless steel substrate.

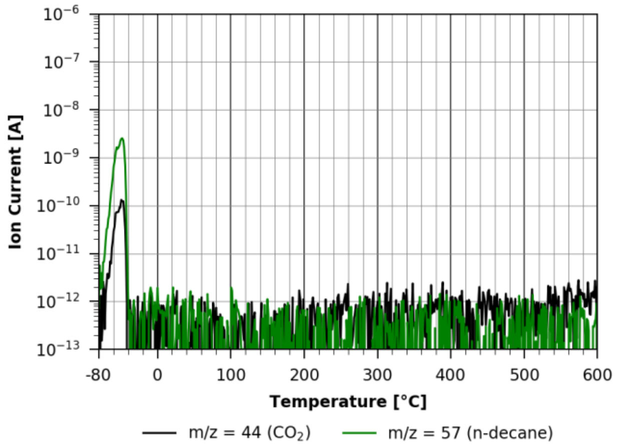

Fig. S4 TPD of n-decane on stainless steel (Henögl, 2020)

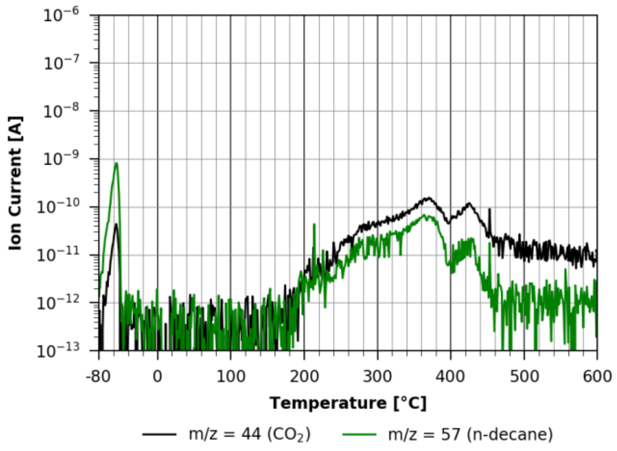

Fig. S5 TPD of n-decane from cellulose 60 min reg. + ASA (Henögl, 2020)

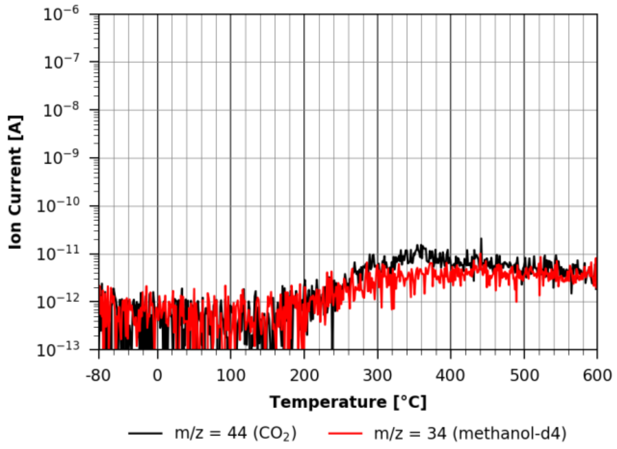

Fig. S6 TPD of methanol-d4 from cellulose 60 min reg. + ASA (Henögl, 2020)

References

Henögl, E. M. (2020) Adsorption and Desorption of Organic Molecules from Thin Cellulose Films Investigated by Temperature Programmed Desorption. Master Thesis, University of Technology, Graz
